# Ubiquitin-like protein FAT10 promotes renal fibrosis by stabilizing USP7 to prolong CHK1-mediated G2/M arrest in renal tubular epithelial cells

**DOI:** 10.18632/aging.204301

**Published:** 2022-09-22

**Authors:** Ying Shao, Wenming Zhang, Dongnian Du, Yi Yu, Qing Li, Xiaogang Peng

**Affiliations:** 1 Queen Mary School, Nanchang University Jiangxi Medical College, Nanchang 330006, Jiangxi Province, China; 2 Department of General Surgery, The Second Affiliated Hospital of Nanchang University, Nanchang 330006, Jiangxi Province, China; 3 Jiangxi Province Key Laboratory of Molecular Medicine, The Second Affiliated Hospital of Nanchang University, Nanchang 330006, Jiangxi Province, China; 4 Department of Urology, The Second Affiliated Hospital of Nanchang University, Nanchang 330006, Jiangxi Province, China; 5 Department of Pathology, The Second Affiliated Hospital of Nanchang University, Nanchang 330006, Jiangxi Province, China

**Keywords:** renal fibrosis, FAT10, cell cycle (G2/M) arrest, checkpoint kinase 1, ubiquitin specific protease 7

## Abstract

Renal fibrosis is the pathological hallmark of chronic kidney disease that is influenced by numerous factors. Arrest of renal tubular epithelial cells (RTECs) in G2/M phase is closely correlated with the progression of renal fibrosis; however, the mechanisms mediating these responses remain poorly defined. In this study, we observed that human leukocyte antigen-F adjacent transcript 10 (FAT10) deficiency abolished hypoxia-induced upregulation of checkpoint kinase 1 (CHK1) expression in RTECs derived from FAT10^+/+^ and FAT10^−/−^ mice. Further investigations revealed that FAT10 contributes to CHK1-mediated G2/M arrest and production of pro-fibrotic cytokines in RTECs exposed to hypoxia. Mechanistically, FAT10 directly interacted with and stabilized the deubiquitylating enzyme ubiquitin specific protease 7 (USP7) to mediate CHK1 upregulation, thereby promoting CHK1-mediated G2/M arrest in RTECs. In animal model, FAT10 expression was upregulated in the obstructed kidneys of mice induced by unilateral ureteric obstruction injury, and FAT10^−/−^ mice exhibited reduced unilateral ureteric obstruction injury induced-renal fibrosis compared with FAT10^+/+^ mice. Furthermore, in a cohort of patients with calculi-related chronic kidney disease, upregulated FAT10 expression was positively correlated with renal fibrosis and the USP7/CHK1 axis. These novel findings indicate that FAT10 prolongs CHK1-mediated G2/M arrest via USP7 to promote renal fibrosis, and inhibition of the FAT10/USP7/CHK1 axis might be a plausible therapeutic approach to alleviate renal fibrosis in chronic kidney disease.

## INTRODUCTION

Renal fibrosis is the most prevalent pathological manifestation of chronic kidney disease (CKD), which is characterized with the renal tubular injury and the accumulation of extracellular matrix (ECM) [1]. For the renal fibrosis, its pathophysiology is complex and contains multiple molecular pathways and cell kinds [2]. Renal tubular epithelial cells (RTECs) exert an essential role in such process. Under severe and long-term injury, RTECs releases various cytokines that influence the mesenchymal fibroblasts in paracrine manner, facilitating their proliferation together with ECM generation [3, 4]. However, the role and specific mechanism of RTECs in facilitating the renal fibrosis is still undefined.

Cell cycle is a significant physiological course and exerts a primary role in tissue damage and repair [5]. During repetitive sublethal injury, RTECs experienced repair and activate complicated pathway networks, including cell proliferation, cell cycle checkpoints, and cell death [6, 7]. In particular, activation of G2/M cell cycle arrest in RTECs is a significant event in the development of renal fibrosis [8]. Many approaches have been used to induce different severities of renal injury; as reported, the RTECs proportion arrested in G2/M phase is evidently raised, and these G2/M phase arrested cells produce profibrotic cytokines, CTGF and TGF-β to generate the renal fibrosis [9]. Conversely, blocking G2/M arrest can attenuate the generation of renal fibrosis and pro-fibrotic cytokines after renal damage [10]. These approaches suggest that G2/M cell cycle arrest in RTECs is critical in controlling renal fibrosis; therefore, a method to delay the G2/M cell cycle arrest may be able to inhibit the development of renal fibrosis.

For the cell proliferation, the development of cell cycle is the prerequisite and is strictly adjusted with negative and positive mediators [11]. Among these mediators, checkpoint kinase 1 (CHK1) is the effector protein kinase, which can modulate the S-phase development and the cell cycle arrest of G2/M phase [12]. When cells are damaged, CHK1 activates the G2/M checkpoint, causes the cell cycle arrest in G2/M phase and promotes the repair of damaged DNA [13]. As reported, the raise of CHK1 phosphorylation is a key variation related to the abrogation of G2/M checkpoint control [14], and CHK1 knockdown reduces the cell cycle G2/M arrest caused by diallyl disulfide in MGC803 (human gastric cancer cell line) [15]. Nevertheless, there is no information on the molecular mechanism or effect of G2/M arrest mediated with CHK1 in renal tubular cells, which causes renal fibrosis.

HLA-F adjacent transcription 10 (FAT10) is a member of the ubiquitin-like (UBL) protein family, which contains two UBL moieties to covalently modify target substrates [16]. Both our and other researches have indicated that in various cells, FAT10 has high upregulation under inflammatory and hypoxic conditions [17–19]. Recently, some researches have displayed that FAT10 is participated in many cellular processes, involving the regulation of cell cycle [20]. Liu et al. showed that FAT10 induced cell cycle progression in hepatocellular carcinoma cells [21]. Previous researches have exhibited that FAT10 is probably an essential component for the pathogenesis of many renal diseases [22, 23]. However, whether FAT10 can promote renal fibrosis by affecting the G2/M phase arrest in RTECs remains the subject of debate.

In this work, we observed that that FAT10 stabilized the CHK1 deubiquitinase USP7 to prolong CHK1-mediated G2/M arrest in RTECs. Furthermore, FAT10 deficiency inhibited extracellular matrix deposition and reduced renal fibrosis after UUO injury in mice. Moreover, our data indicated that the FAT10/USP7/CHK1 axis is positively associated with renal fibrosis in kidneys obtained from patients with calculi-related CKD.

## MATERIALS AND METHODS

### Mice and surgical protocol

Wild-type C57BL/6 (FAT10^+/+^) mice were obtained from the Model Animal Research Center of Nanjing University. Profit from Model Animal Research Center of Nanjing University, the CRISPR-Cas9 technology was utilized for creating FAT10-knockout mice (FAT10^−/−^) on a C57BL/6 background. Knockout of FAT10 was confirmed by genotyping and Western blotting (Supplementary Figure 1A and 1B). And, the Western blotting results showed that FAT10 was expressed in mouse thymus, spleen, kidney and heart but not in mouse brain, whereas this expression pattern was abolished in FAT10^−/−^ mice (Supplementary Figure 1C).

A left kidney UUO model was performed as previously described [24]. Shortly, mice aged 8–10 weeks old were anesthetized through intraperitoneal injection of 5% pentobarbital sodium (50 mg/kg). The incision of left upper quadrant was 1.5 cm, and the left ureter was ligated with 4–0 silk suture. In the sham-operated mice, ureters were exposed but not ligated. All of the experimental steps were implemented based on NIH guidelines for the Care and Use of Laboratory Animals and authorized through the Animal Ethics Committee of Nanchang University.

### Cell culture and treatment

Primary RTECS were separated from the renal cortex of mice aged 3–4 weeks (FAT10^−/−^ and FAT10^+/+^) as described formerly [25]. HK-2, the human proximal tubular epithelial cell line, was obtained from the Cell Bank of the Chinese Academy of Sciences. The cells were cultivated under the normoxic environments (95% humidity, 5% CO_2_, 21% O_2_, 37°C) or under hypoxic environments (95% humidity, 5% CO_2_, 1% O_2_, under a temperature of 37°C) in a hypoxia incubator (Thermo Fisher Scientific, USA).

### Liquid chromatography with tandem mass spectrometry (LC–MS/MS) analysis

The analysis of LC-MS/MS was implemented as previously mentioned [26]. In brief, whole lysates of RTECs were extracted from FAT10^+/+^ or FAT10^−/−^ RTECs and analyzed by label-free tandem mass tag. Subsequently, with the aid of Shanghai Applied Protein Technology Co., Ltd, the analysis of acquired samples was carried out through LC-MS/MS. In addition, MaxQuant software version 1.3.05 (Max Planck Institute of Biochemistry in Martinsried, Germany) was applied for analyzing the data.

### Quantitative real-time PCR (qRT–PCR)

Real-Time PCR Detection System (Thermo Fisher Scientific, USA) together with SYBR Green PCR Master Mix (Takara, Japan) were conducted for qRT-PCR. The primer sets utilized for the SYBR green analysis of human CHK1, USP7 and FAT10 were below: FAT10, 5′-CTCTGGTTTCTGGCCCCTTG-3′ and 5′-CCATTCCTCGGAACGGACAT-3′. USP7, 5′-CAGAGATGGCTGGGAACCAC-3′ and 5′-TTGTGTCCATCACTCAGGGC-3′. CHK1, 5′-TTGGTTGACTTCCGGCTTTCT-3′ and 5′-GTCCGATCATGTGGCAGGAA-3′. GAPDH, 5′-CATACCAGGAAATGAGCTTGAC-3′ and 5′-AACAGCGACACCCACTCCTC-3′. The housekeeping gene GAPDH was used as control.

### Overexpression constructs and shRNA plasmids

Genepharma (Shanghai, China) produced the eukaryotic expression vector pcDNA3.1 encoding Flag-CHK1, Flag-USP7 or His-FAT10 either and plasmids encoding shRNAs targeting USP7 or FAT10. Subsequently, based on the instructions of manufacturer, HK-2 cells could be transfected through these shRNA plasmids or overexpression constructs utilizing Lipofectamine 3000 (Invitrogen, USA).

### Cell cycle distribution analysis

After treatment, the cells were washed for two times utilizing ice-cold PBS, gathered, fixed in ethanol (70%) at 4°C, and next inoculated under a temperature of 37°C in propidium iodide (50 μg/ml, Sigma-Aldrich) and DNasefree RNase A (1 mg/ml, Sigma-Aldrich) for 60 minutes. Flow cytometry (FACS Calibur, BD Biosciences) was exploited for analyzing the distribution of cell cycle.

### Western blot, Co-immunoprecipitation (Co-IP), *in vivo* ubiquitination assay and GST pull-down assays

As formerly described, the Co-IP, western blotting, GST pull-down as well as *in vitro* ubiquitination assays were implemented [18, 25]. For the Western blot analysis, cells or tissues were dissolved in Tris (20 mM, with a pH of 7.8), NaCl (150 mM), 0.1% Triton X-100 with protease inhibitors (phenylmethylsulphonyl fluoride (PMSF) 100 mM, 1 μM pepstatin and 5 μg/ml aprotinin). Subsequently, SDS/PAGE was applied to separate the equal amounts of cell lysates, which were electrotransferred to the membranes of polyvinylidene fluoride (Millipore, USA) and blocked in skim milk (5%). Through using the specific primary antibodies, and then the proper secondary antibodies conjugated with HRP, the membranes were immunoblotted. The immunoreactive bands were observed using a chemiluminescence kit with high sensitivity. The following antibodies were used: antibodies against FAT10 (1:1000, Santa cruz, sc-393630), USP7 (1:1000, Abcam, ab108931), CHK1 (1:1000, CST, #2360), CDKN1A (1:1000, Abcam, ab102013), TGF-β (1:1000, Abcam, ab215715), and CTGF (1:1000, Abcam, ab209780), Flag (1:1000, Sigma, F1804), His (1:1000, Abmart, M30111), ubiquitin (1:1000, Abcam, ab140601) and Tubulin (1:1000, Abcam, ab7291).

### Cohort of patients with chronic tubulointerstitial disease

There are 30 patients with biopsy proven chronic tubulointerstitial disease (cases resulted from CKD associated with calculi) and 3 normal human kidneys in patients with renal angiomyolipomas were included in the study. All the patients provided the written informed consent, and this study was authorized through the Ethics and Research Committees of the Second Affiliated Hospital of Nanchang University.

### Histopathology and immunohistochemical (IHC) staining

With routine procedures, the kidney sections were generated at thickness of 4 mm. Picrosirius Red reagents and Masson’s Trichrome were utilized for staining the sections Besides, such sections were inoculated through primary antibodies overnight under a temperature of 4°C and subsequently with the secondary antibodies. Nikon light microscope was applied for taking the images, and the analysis of staining intensity was implemented with Nikon software (Nikon, USA).

### Statistical analysis

All of the data are described as mean ± SEM. ANOVA and Student-Newman-Keuls tests were employed for the comparison of the mean of multiple groups. GraphPad Prism version 6.0 software (USA) was applied for the analyses. *P* < 0.05 was considered statistically significant.

## RESULTS

### FAT10 regulates CHK1 protein expression in RTECs exposed to hypoxia

**Figure 1:**
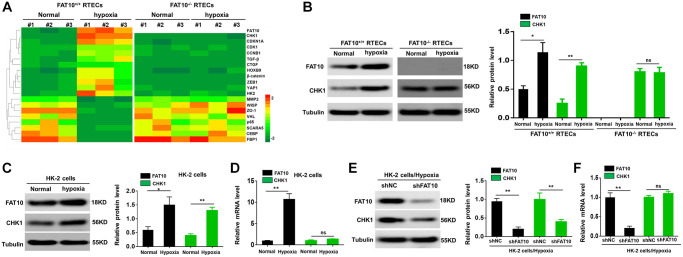
**Hypoxia-induced CHK1 upregulation in RTECs is depend on FAT10.** (**A**) Upon hypoxia treatment for 24 h, mass spectroscopic analysis was performed to detect protein expression in RTECs from FAT10^+/+^ mice (*n* = 3) and FAT10^−/−^ mice (*n* = 3). (**B**) Determination (left) and quantification (right) of the FAT10 and CHK1 protein levels in FAT10^+/+^ RTECs or FAT10^−/−^ RTECs after hypoxia injury. Tubulin was used as a loading control. ^*^P < 0.05, ^**^P < 0.01. (**C**) Determination (left) and quantification (right) of the FAT10 and CHK1 protein levels in HK-2 cells following treatment with hypoxia or without hypoxia. ^*^P < 0.05, ^**^P < 0.01. (**D**) The protein and mRNA levels of FAT10 and CHK1 in HK-2 cells following treatment with hypoxia or without hypoxia. ^**^P < 0.01. (**E**) Determination (left) and quantification (right) of the FAT10 and CHK1 protein levels in HK-2 cells transfected with shFAT10 following hypoxic injury. ^**^P < 0.01. (**F**) Upon hypoxia treatment for 24 h, the mRNA levels of FAT10 and CHK1 in HK-2 cells transfected with shFAT10. ^**^P < 0.01.

Hypoxia has been considered as a significant microenvironment element in the occurrence and development of renal fibrosis [27]. To investigate the relationship between FAT10 and CHK1 in RTECs, we initially isolated and cultured primary RTECs from FAT10^+/+^ and FAT10^−/−^ mice, and exposed them to hypoxia condition for 24 h. Subsequent analysis of RTEC lysates by LC-MS/MS revealed that FAT10 and CHK1 proteins were abnormally increased in FAT10^+/+^ RTECs; however, these responses were diminished in FAT10^−/−^ RTECs (Figure 1A). The western blot data further demonstrated that hypoxia raised the CHK1 and FAT10 protein expression and in FAT10^+/+^ RTECs in contrast to the control groups, but the protein level of CHK1 remained unchanged in FAT10^−/−^ RTECs after treatment with hypoxia (Figure 1B). Thus, hypoxia-induced upregulation of CHK1 was dependent on FAT10.

Next, we found that FAT10 could regulate the expression of CHK1 in HK-2 cells exposed to hypoxic injury. Under hypoxic conditions, the protein and mRNA levels of FAT10 were upregulated in HK-2 cells, and the protein level of CHK1 was also upregulated; however, the mRNA level of CHK1 was not changed in HK-2 cells (Figure 1C and 1D). Moreover, the protein level of CHK1 was significantly decreased in FAT10-silenced HK-2 cells exposed to hypoxia; however, the mRNA level of CHK1 was not changed in FAT10-knockdown HK-2 cells (Figure 1E and 1F). Overall, these findings indicated that FAT10 could regulate the protein expression of CHK1 in RTECs exposed to hypoxia.

### FAT10 impacted G2/M arrest in RTECs by regulating the CHK1 expression under hypoxia

**Figure 2:**
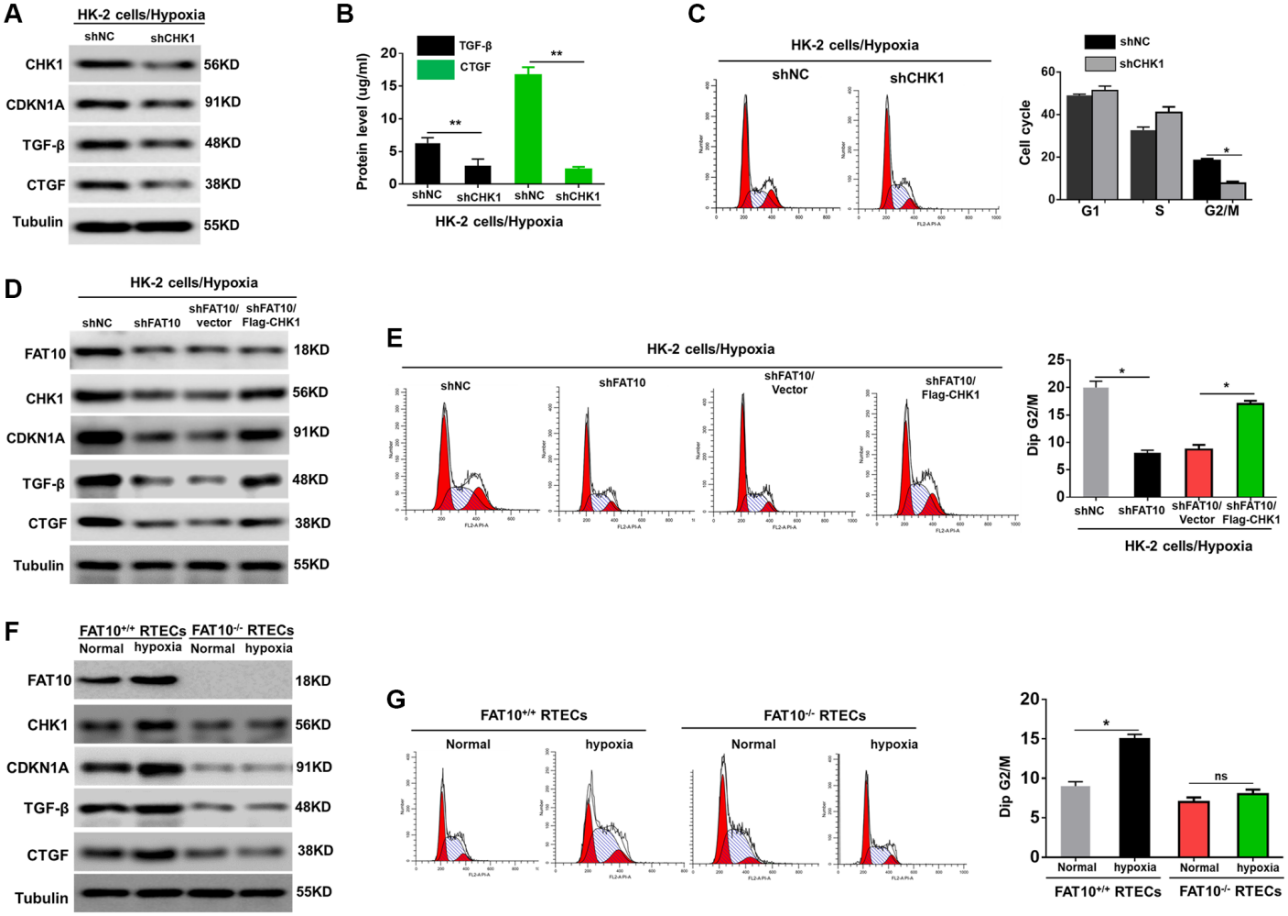
**FAT10 is required for CHK-1-mediated G2/M arrest in RTECs under hypoxia treatment.** (**A**) Western blotting showing the protein expression of CHK1, CDKN1A, TGF-β and CTGF in CHK1-silencing HK-2 cells following hypoxia injury. Tubulin was used as a loading control. (**B**) TGF-β and CTGF in the culture supernatants was measured in culture supernatants by ELISA assay. ^**^P < 0.01. (**C**) Detection for cell cycle of CHK1-silencing HK-2 cells following hypoxia injury. Results are expressed as peak diagram (left) and calculated distribution for cells in G0/G1, S and G2/M phases (right). ^*^P < 0.05. (**D**) Upon hypoxia treatment, western blotting of FAT10, CHK1, CDKN1A, TGF-β and CTGF in HK-2 cells stably transfected with shFAT10 in the presence or absence of Flag-CHK1. (**E**) Detection for cell cycle of FAT10-silencing HK-2 cells in the presence or absence of Flag-CHK1 following hypoxia injury. Results are expressed as peak diagram (left) and calculated distribution for cells in G0/G1, S and G2/M phases (right). ^*^P < 0.05. (**F**) Western blotting showing the protein expression of FAT10, CHK1, CDKN1A, TGF-β and CTGF in FAT10^+/+^ RTECs and FAT10^−/−^ RTECs following treatment with hypoxia or without hypoxia. (**G**) Detection for cell cycle of FAT10^+/+^ RTECs and FAT10^−/−^ RTECs following treatment with hypoxia or without hypoxia. Results are expressed as peak diagram (left) and calculated distribution for cells in G0/G1, S and G2/M phases (right). ^*^P < 0.05.

Various reports have demonstrated that G2/M cell cycle arrest mainly occurs following the activation of CHK1 [12, 15]. Previous researches have confirmed that FAT10 is participated in regulating the cell cycle [20, 21]. Thus, we speculated that FAT10 could impact G2/M arrest in RTECs by regulating the expression of CHK1 when exposed to hypoxia. To test this hypothesis, we first analyzed the expression of the two markers of G2/M cell cycle arrest CHK1 and cyclin dependent kinase inhibitor 1A (CDKN1A) in HK-2 cells after treatment with hypoxia. As shown in Supplementary Figure 2A, hypoxic injury triggered increased expression of CHK1 and CDKN1A in HK-2 cells. Our findings also displayed that in HK-2 cells, the profibrotic cytokines (including CTGF and TGF-β) were raised under hypoxic environments (Supplementary Figure 2A). We next examined the CTGF and TGF-β contents in the supernatants of the hypoxic HK-2 cells. Our outcomes revealed that both CTGF and TGF-β were remarkably enriched in conditioned media (Supplementary Figure 2B). Consistently, hypoxia increased the proportion of cells in G2/M (Supplementary Figure 2C). Moreover, when CHK1 was silenced, hypoxic injury failed to upregulate CDKN1A, TGF-β, and CTGF (Figure 2A and 2B). Quantitative analysis exhibited that the number of cells in G2/M phase was evidently reduced in cells with CHK1 knockout in contrast to control cells (Figure 2C). These findings suggested that CHK1 exert an essential role in G2/M cell cycle arrest in RTECs exposed to hypoxia.

Next, we investigated whether FAT10 influenced G2/M arrest of tubular cells when exposed to hypoxia by upregulating CHK1. The results indicated that overexpression of CHK1 rescued the downregulation of CHK1, CDKN1A, TGF-β, and CTGF in FAT10-silenced HK-2 cells induced by hypoxia injury (Figure 2D, Supplementary Figure 3A and 3B). Consistent with these results, re-expression of CHK1 in FAT10-silenced HK-2 cells rescued the prolonged G2/M arrest in response to hypoxia treatment (Figure 2E). Moreover, we complemented studies *in vitro* by evaluating the effects FAT10 on primary RTECs. Hypoxic treatment increased CHK1, CDKN1A, TGF-β, and CTGF in FAT10^+/+^ RTECs; however, the hypoxia-mediated induction of these responses was almost completely abolished in FAT10^−/−^ RTECs (Figure 2F, Supplementary Figure 3C and 3D). Consistently, the accumulation of FAT10^+/+^ RTECs in G2/M was also abolished in primary FAT10^−/−^ RPTCs under hypoxic injury (Figure 2G). Thus, these data suggested that FAT10 mediated prolonged G2/M arrest by regulating the expression of CHK1 following exposure to hypoxia.

### FAT10 stabilizes the CHK1 deubiquitinase USP7 by antagonizing its ubiquitination level

**Figure 3:**
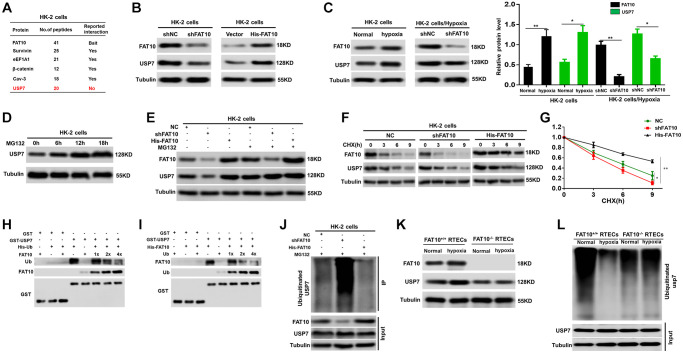
**FAT10 interacting with USP7 and stabilising USP7 expression.** (**A**) A partial list of FAT10-associated proteins were indicated by immunoprecipitation-mass spectrometry. (**B**) Protein levels of FAT10 and USP7 in FAT10-overexpressing or FAT10-silenced HK-2 cells were detected by western blotting. Tubulin was used as a loading control. (**C**) Determination (left) and quantification (right) of FAT10 and USP7 protein levels in HK-2 cells or FAT10-silenced HK-2 cells following treatment with hypoxia or without hypoxia. ^*^P < 0.05, ^**^P < 0.01. (**D**) Western blot showing USP7 protein levels in HK-2 cells following treatment with 10 μM MG132 at different times. (**E**) HK-2 cells transduced with shFAT10 or Flag-FAT10 were treated with MG132. Cells were collected at 6 h and immunoblotted with the antibodies indicated. (**F** and **G**) Representative (**F**) and quantitative (**G**) results of USP7 protein level in FAT10-overexpression or FAT10-silencing cells. The cells were treated with cycloheximide (CHX, 100 μg/ml) for indicated time points were subjected to western blot analysis. The degradation rate of USP7 protein was calculated according to the ratio of USP7/tubulin. The quantification data represent mean ± SD from three independent experiments and were statistically analyzed with Student’s *t*-test, ^*^P < 0.05, ^**^P < 0.01. (**H** and **I**) Competitive binding of USP7 was analyzed in a GST-pull down experiment. HEK-293 T cells were transfected with the indicated constructs and lysed for IP using anti-His beads to detect GST binding. (**J**) Knockdown or exogenous expression of FAT10 in HK-2 cells altered the ubiquitination of USP7. The cells in each group were treated with MG132. (**K**) Western blotting showing the protein expression of FAT10 and USP7 in FAT10^+/+^ RTECs and FAT10^−/−^ RTECs following treatment with hypoxia or without hypoxia. (**L**) Ubiquitinated USP7 in in FAT10^+/+^ RTECs and FAT10^−/−^ RTECs following treatment with hypoxia or without hypoxia. The cells in each group were treated with MG132.

We investigated the mechanism that FAT10 modulate the expression of CHK1 in RTECs. Surprisingly, Co-immunoprecipitation (IP) assays indicated that FAT10 could not bind with CHK1 in HK-2 cells (Supplementary Figure 4). The researches have displayed that the deubiquitinase USP7 can modulate the CHK1 expression [28, 29]; therefore, we speculated that FAT10 may influence the expression of CHK1 by regulating USP7. To prove this hypothesis, we initially screened FAT10-interacting proteins using mass spectrometric analysis. Interestingly, we observed that FAT10 interacts with USP7 (Figure 3A). Through utilizing endogenous USP7 and FAT10 antibodies in HK-2 cells, Co-IP analysis in-depth proved the interaction between USP7 and FAT10 (Supplementary Figure 5A). Besides, the confocal assays revealed that USP7 and FAT10 colocalization was significant in HK-2 cells (Supplementary Figure 5B). Additionally, it can be observed that the USP7 protein level was attenuated after knockdown of FAT10, but increased with FAT10 overexpression (Figure 3B). Moreover, our outcomes suggested that the protein level of USP7 was obviously elevated in HK-2 cells under hypoxic conditions, but reduced in FAT10-knockdown cells (Figure 3C). However, the mRNA levels of USP7 were not changed in FAT10-silenced or FAT10-overexpressing HK-2 cells with or without hypoxic injury (Supplementary Figure 6). Therefore, these findings suggested that FAT10 can regulate the protein expression of USP7 in RTECs.

Subsequently, the mechanism that FAT10 modulates USP7 was analyzed. The former researches have reported that the USP7 protein stability is modulated via the UPS [30]. Furthermore, our studies have confirmed that FAT10 can antagonize ubiquitination of substrates and stabilize the expression of substrates in different cells [18, 31]. Thus, we speculated that FAT10 could affect the ubiquitination level of USP7, subsequently affecting its expression. Our outcomes proved our assumption as expected. At first, we observed that USP7 was degraded through UPS and FAT10 was participated in the USP7 degradation in HK-2 cells (Figure 3D and 3E). Second, a degradation dynamics assay revealed that overexpression of FAT10 in HK-2 cells led to a pronounced increase in USP7 protein stability, whereas FAT10 silencing in HK-2 cells reduced USP7 stability (Figure 3F and 3G). Third, through GST pull-down analysis, it can be found that in HEK 293T cells, Ub and FAT10 competed to bind with USP7 (Figure 3H and 3I). Eventually, *in vivo* ubiquitination together with Co-IP assays displayed that in HK-2 cells, reduction of FAT10 raised the USP7 ubiquitination level, while FAT10 overexpression reduced the USP7 ubiquitination level (Figure 3J). Furthermore, hypoxic treatment increased the protein level of USP7 and reduced the ubiquitination level of USP7 in FAT10^+/+^ RTECs; however, the hypoxia-induced response described above was almost completely abolished in FAT10^−/−^ RTECs (Figure 3K and 3L). In summary, FAT10 directly interacts with USP7 and stabilises USP7 expression by antagonizing its ubiquitination level in RTECs.

### FAT10 influences the expression of CHK1 via action on USP7 to regulate G2/M cell cycle arrest in RTECs

**Figure 4:**
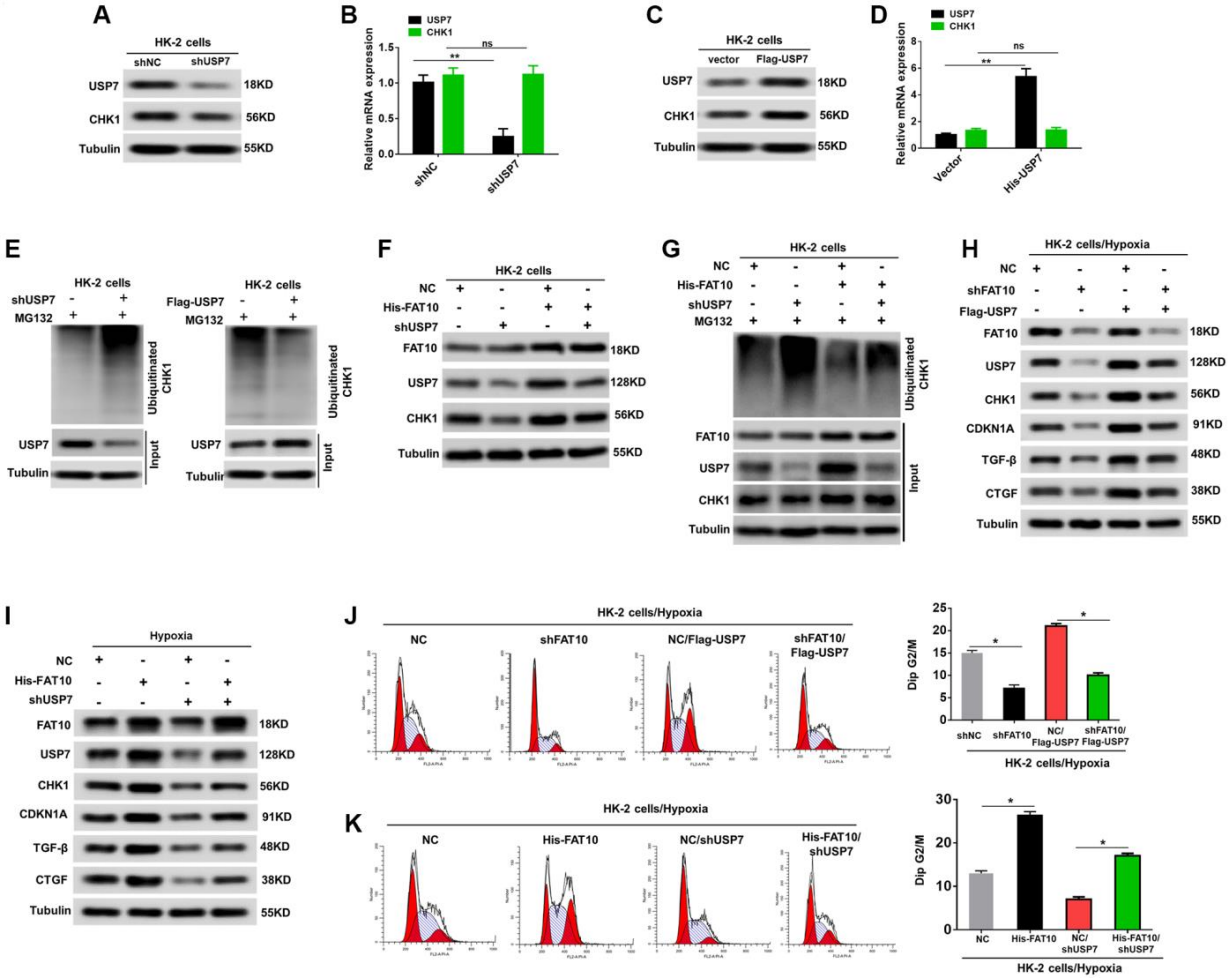
**FAT10 regulates CHK1 expression through USP7.** (**A** and **B**) Western blotting and qRT-PCR analyses of CHK1 expression levels in HK-2 cells stably transfected with shNC or shUSP7. (**C** and **D**) Western blotting and qRT-PCR analyses of CHK1 expression levels in HK-2 cells stably transfected with control vector or Flag-USP7. ^**^P < 0.01. Tubulin was used as a loading control. (**E**) Knockdown or exogenous expression of USP7 altered the ubiquitination of CHK1 in HK-2 cells. The cells in each group were treated with MG132. (**F**) Western blotting of FAT10, CHK1 and USP7 in HK-2 cells stably transfected with Flag-FAT10 in the presence or absence of shUSP7. (**G**) Ubiquitinated CHK1 in HK-2 cells stably transfected with Flag-FAT10 in the presence or absence of shUSP7. The cells in each group were treated with MG132. (**H** and **I**) Upon hypoxia treatment, western blotting of FAT10, CHK1, CDKN1A, TGF-β and CTGF in FAT10-silencing HK-2 transfected with Flag-USP7 (**H**) or in FAT10-overexpression HK-2 transfected with shUSP7 (**I**). (**J** and **K**) Detection for cell cycle of FAT10-silencing HK-2 transfected with Flag-USP7 (**J**) or FAT10-overexpression HK-2 transfected with shUSP7 (**K**) following hypoxia injury. Results are expressed as peak diagram (left) and calculated distribution for cells in G2/M phases (right). ^*^P < 0.05.

Next, we explored whether FAT10 influences the expression of CHK1 via USP7, thus affecting the G2/M cell cycle arrest in RTECs. Previous research proved that USP7 directly interacts with CHK1, and deubiquitylates and stabilizes the protein [28]. We initially examined whether USP7 regulates the CHK1 expression level via attenuating the CHK1 ubiquitination level in HK-2 cells. As shown in Figure 4A–4D, the protein level of CHK1 was decreased with USP7 knockdown, but increased with USP7 overexpression; however, the mRNA level of CHK1 showed no difference after USP7 dysregulation in HK-2 cells. Further, our data also demonstrated that the USP7 knockdown can raise the level of CHK1 ubiquitination and vice versa (Figure 4E). Additionally, the outcomes suggested that the FAT10 overexpression can raise the expression of CHK1, whereas USP7 knockdown abolished this increase (Figure 4F). Meanwhile, the results revealed an increased the poly-ubiquitination of CHK1 in HK-2 cells with knockdown of endogenous USP7, even following FAT10 overexpression (Figure 4G). Thus, these results demonstrated that FAT10 could influence the expression of CHK1 through USP7.

We further performed rescue experiments to determine whether FAT10 influenced CHK1-mediated G2/M arrest of RTECs by USP7 enhancement. As shown in Figure 4H, overexpression of USP7 rescued USP7, CHK1, CDKN1A, TGF-β, and CTGF expression in FAT10-silenced HK-2 cells subjected to hypoxic injury. Consistent with these results, restoration of USP7 in FAT10-silenced HK-2 cells rescued the prolonged G2/M arrest observed in response to hypoxia treatment (Figure 4J). Conversely, USP7 knockdown inhibited the increase in USP7, CHK1, CDKN1A, TGF-β, and CTGF expression, and significantly reduced G2/M arrest in the HK-2 cells with FAT10 overexpression under the hypoxic environments (Figure 4I and 4K). Collectively, FAT10 is involved in CHK1-mediated G2/M arrest in RTECs by regulating USP7 expression.

### FAT10 deficiency alleviated renal fibrosis after UUO injury

**Figure 5:**
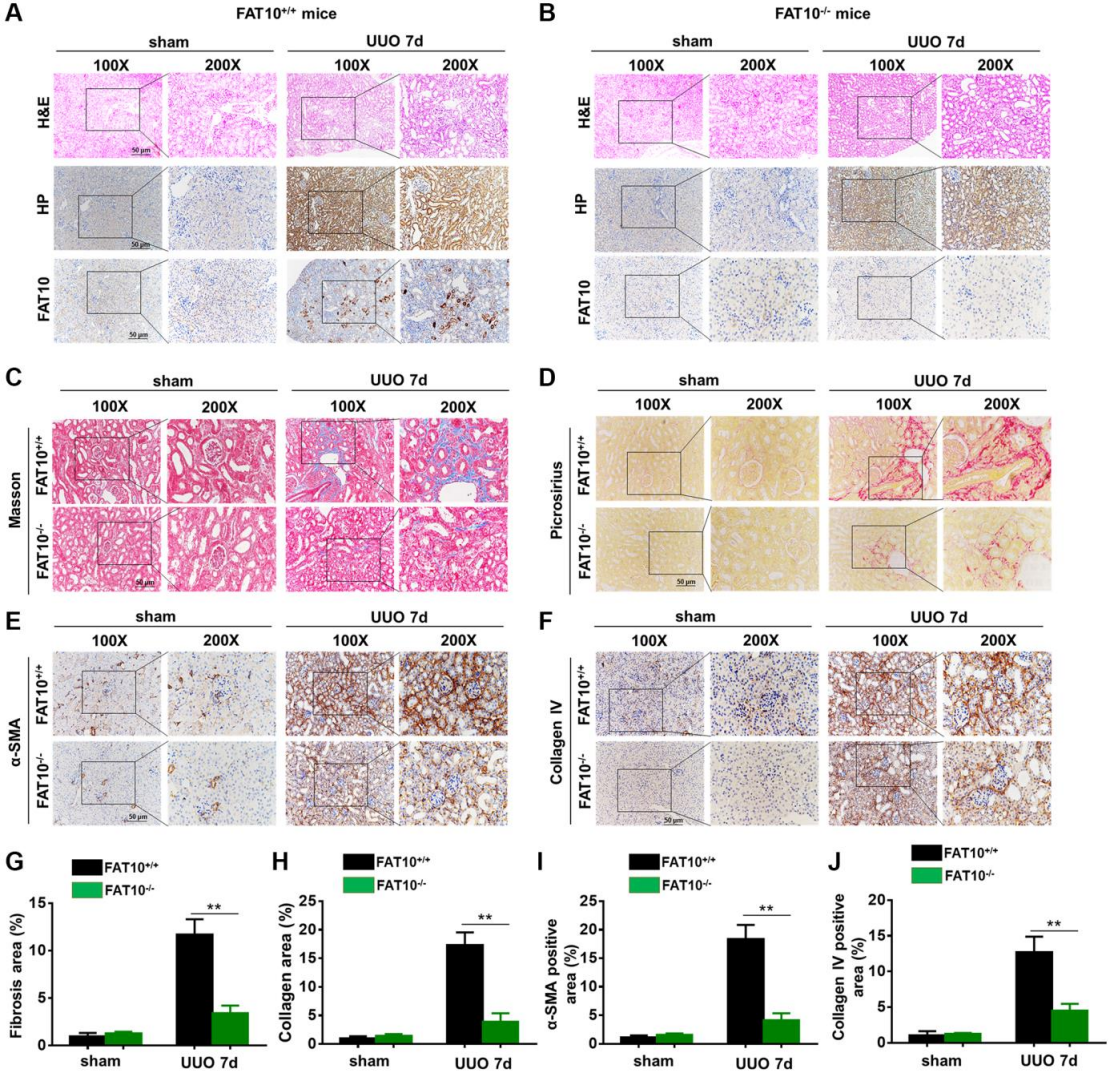
**UUO-induced kidney fibrosis was suppressed in FAT10-deficient mice.** (**A** and **B**) Representative HP and FAT10 staining in the kidneys from FAT10^+/+^ (**A**) and FAT10^−/−^ (**B**) mice subjected to either UUO or sham operation; scale bar = 50 μm. (**C** and **D**) Representative Masson’s trichrome (**C**) and picrosirius red staining (**D**) of kidney sections from FAT10^+/+^ and FAT10^−/−^ mice with or without UUO for 7 days. (**E** and **F**) Immunohistochemistry of protein expression of α-SMA (**E**) and Collagen IV (**F**) in obstructed kidneys from FAT10^+/+^ and FAT10^−/−^ mice subjected to either UUO or sham operation. (**G** and **H**) Bar graph (right) shows quantification of fibrotic areas in histological sections; ^**^P < 0.01 versus FAT10^+/+^ mice at the same time point; *n* = 6. (**I** and **J**) Bar graph shows quantification of areas of positive cells; ^**^P < 0.01 versus FAT10^+/+^ mice at the same time point; *n* = 6.

To determine the role of FAT10 in renal fibrosis, FAT10^+/+^ and FAT10^−/−^ mice were subjected by unilateral ureteric obstruction (UUO), a well-established renal fibrosis animal model [32]. Compared with the sham group, the level of hypoxia (measured by staining for the hypoxic marker HP) was increased in obstructed kidneys from FAT10^+/+^ and FAT10^−/−^ mice, and the overexpression of FAT10 was observed in obstructed kidneys from the FAT10^+/+^ mice by immunohistochemistry analysis (Figure 5A and 5B). Consistently, the expression of FAT10 were increased in qRT-PCR and Western blot analyses in the obstructed kidneys following UUO injury (Supplementary Figure 7A and 7B).

Next, we compared the severity of renal fibrosis in FAT10^+/+^ and FAT10^−/−^ UUO-injured mice for 7 days following injury. As revealed with through Picrosirius red together with Masson trichrome staining, FAT10^+/+^ mice formed evident ECM deposits in the obstructed kidneys, and such responses were significantly decreased in FAT10^−/−^ mice (Figure 5C, 5D, 5G and 5H). Similar results were observed when kidney sections were immunostained with α-smooth muscle actin antibody and collagen IV (molecular signatures of myofibroblasts) in the obstructed kidneys (Figure 5E, 5F, 5I and 5J). In addition, we found that the level of pro-fibrotic cytokines (TGF-β and CTGF) were elevated in obstructed kidneys from FAT10^+/+^ mice, whereas these responses were significantly reduced in FAT10^−/−^ mice (Supplementary Figure 8). Thus, these results suggested that FAT10 deficiency reduces renal fibrosis in the kidneys following UUO injury.

### Correlation among FAT10, USP7 and CHK1 expression in renal fibrosis of patients with fibrotic kidney disease

**Figure 6:**
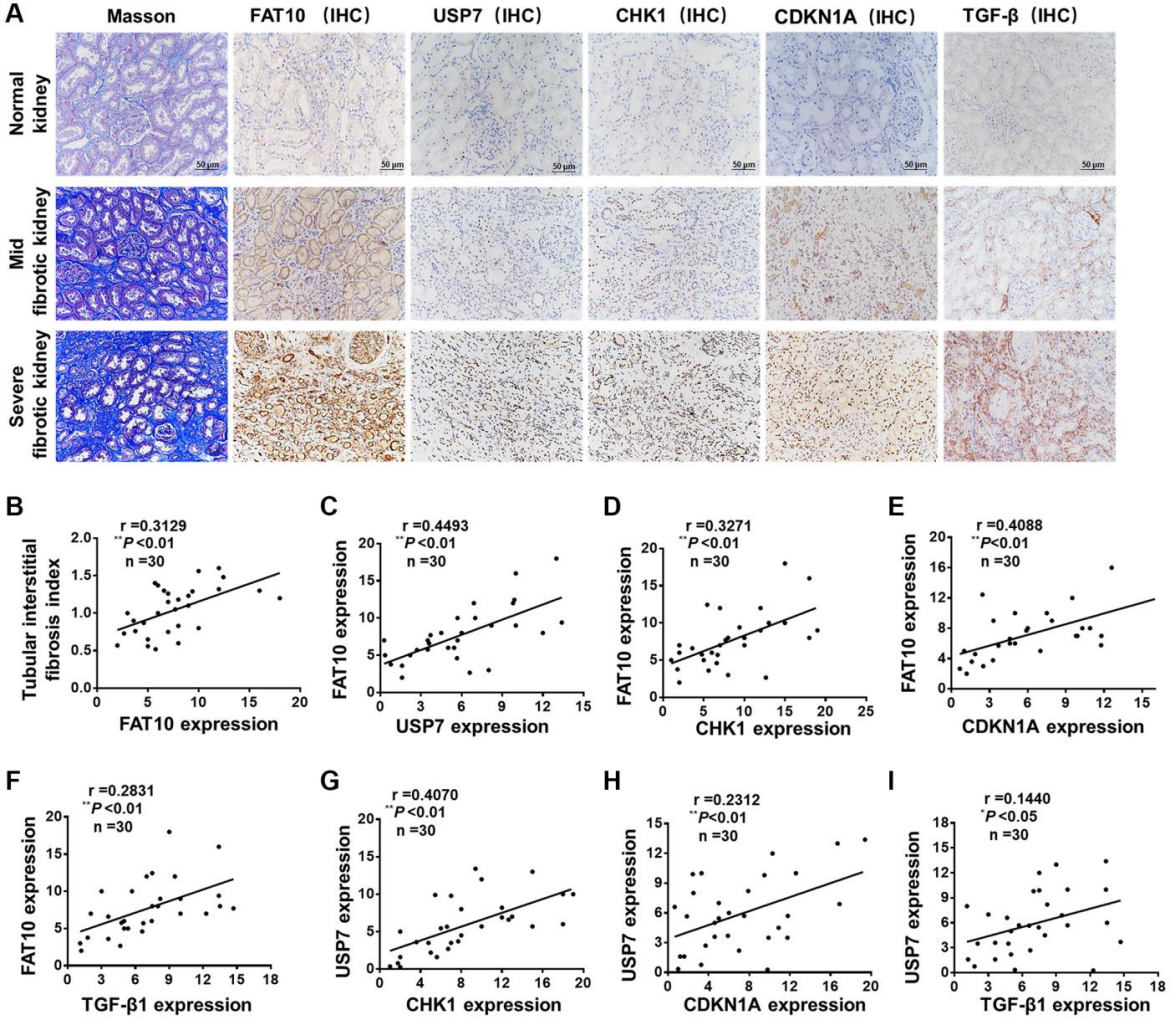
**FAT10 was up-regulated and positively correlated with renal fibrosis in patients with calculi related chronic kidney disease.** (**A**) Representative photos of renal sections from normal kidney and biopsy samples from patients with calculi related chronic kidney disease. IHC, immunohistochemistry; scale bar = 50 μm. (**B**) The expression of renal FAT10 at initial biopsy positively correlated with the tubular interstitial fibrosis index in a partial correlation analysis. (**C**–**F**) Scatter plots show a positive correlation between FAT10 and USP7, CHK1, CDKN1A, TGF-β, respectively. (**G**–**I**) The statistical analysis data show that the expression level of USP7 was positively correlated with the expression level of CHK1, CDKN1A and TGF-β. ^*^P < 0.05, ^**^P < 0.01.

For assessing the impact of FAT10 on the human renal TIF pathogenesis, we implemented a cross-sectional analysis for the correlation between TIF and the expression of FAT10 in 30 patients with CKD associated with calculi, a cohort with the potentiality to develop renal fibrosis. Among these patients, 53.3% (16 cases) had a TIF index ≥1 (Figure 6A and 6B). IHC analysis revealed that FAT10 was not appreciably observed in normal human kidneys in patients with renal angiomyolipomas, and the expression of FAT10 was significantly raised in the renal samples from the patients with CKD related to calculi (Figure 6A). We observed that USP7, CHK1, CDKN1A and TGF-β were also up-regulated in the renal biopsy samples of patients compared with normal human kidneys (Figure 6A). Partial correlation analysis exhibited an evident positive relation between TIF index and the expression levels of FAT10 (Figure 6B). Moreover, in the kidney, the expression levels of FAT10 had positive relation with the expression of CHK1, USP7, TGF-β and CDKN1A in the partial correlation analysis (Figure 6C–6F). Besides, based on the statistical analysis, there exist a positive association between USP7 expression level and TGF-β, CDKN1A and CHK1 expression levels in kidney tissues (Figure 6G–6I). Thus, these results suggested that the FAT10/USP7/CHK1 axis was activated in kidneys in patients with calculi-related CKD, thereby leading to renal fibrosis.

## DISCUSSION

FAT10 is a ubiquitin-like modifier that targets proteins for degradation following recognition by the 26S proteasome [16, 33]. This enzyme has been shown to influence many cellular processes, including cell cycle regulation [20]. In recent, the influence of FAT10 on the malignant tumours is investigated extensively [34], while other studies reported that the FAT10 is participated in renal disease pathogenesis [22, 23]. However, there is currently no information on the impact of FAT10 on the renal fibrosis. In this work, we discovered that FAT10 prolongs CHK1-mediated cell cycle G2/M arrest in RTECs by stabilizing USP7 when exposed to hypoxic injury. Using mice UUO models, we demonstrated that FAT10 was abundantly expressed in the obstructed kidney, and deletion of FAT10 obviously reduced UUO injury-induced renal fibrosis. Additionally, the FAT10/USP7/CHK1 axis was activated in kidneys from patients with calculi-related CKD, thereby leading to renal fibrosis. As is known to all, this is the first report on the potential mechanism and effect of FAT10 on the renal fibrosis.

Emerging evidence from studies in cultured kidney cells and experimental animals suggest that G2/M arrest of tubular cells is the most critical pathophysiological step during the development of renal fibrosis [35, 36]. Cell cycle G2/M arrest is tightly regulated by cell cycle mediators, of which CHK1 contributes to G2/M cell cycle arrest [12, 37]. However, CHK1 is an upstream regulator for the G2/M arrest, and its impact on the renal fibrosis has not been demonstrated. Here, we showed that CHK1 was upregulated in hypoxia-treated-HK-2 cells, and CHK1 deficiency induced HK-2 cells to lower G2/M arrest, which coincided with a marked decrease in the levels of TGF-β and CTGF. These findings suggest the existence of a crosstalk between CHK1-mediated G2/M arrest and renal fibrosis. Additionally, we found that FAT10 could impact hypoxia-induced G2/M cell cycle arrest in RTECs by regulating the expression of CHK1, while FAT10-deficient RTECs exhibit prolonged CHK1-mediated G2/M arrest under hypoxic conditions.

**Figure 7:**
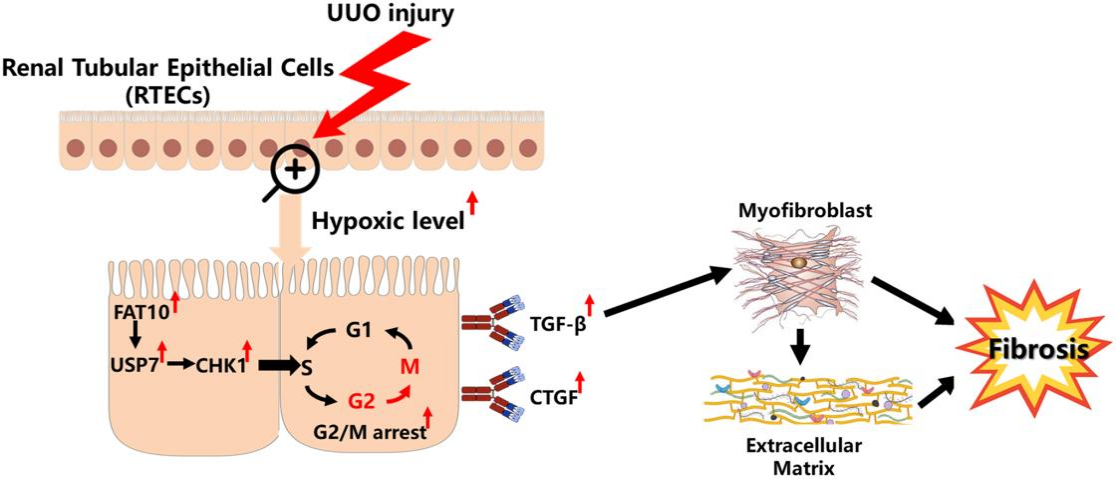
**Model summarizing the role of FAT10 in renal fibrosis response to UUO injury.** Upon UUO injury, FAT10 up-regulation stabilizes USP7 expression, thereby leading to CHK1-mediated G2/M arrest in RTECs, which further drives fibrogenic responses.

Previous studies have demonstrated that CHK1 is strictly modulated with the UPS [38, 39]. For instance, Cul4-involving E3 ubiquitin ligase complexes target CHK1 for the degradation and polyubiquitylation in cells experiencing replication stress [40]. The deubiquitylase USP7 directly regulates CHK1 protein levels by cleaving the poly-ubiquitination chain [28, 29]. Here, our data also indicate that USP7 can regulate the expression level of CHK1 by reducing its ubiquitination level in RTECs. Although USP7 exerts an essential role in a variety of cellular bio-processes, little is known about the cellular modulation of USP7 stability. Previous research reported that the USP7 protein stability is modulated with the ubiquitin proteasome pathway in the cancer cells [30]. However, the mechanism by which USP7 is regulated in RTECs remains unclear. Like ubiquitin, FAT10 is a prevalent signal for the proteasomal degradation [16]. But, our findings confirmed that the function of FAT10 goes beyond the degradation of protein, and FAT10 stabilizes particular substrates via antagonizing their ubiquitination [18, 26]. In line with the previous studies, we also observed that FAT10 stabilizes USP7 by reducing the ubiquitination level of USP7 in RTECs. This summary is on the basis of these observations. First of all, we determined a direct interaction between USP7 and FAT10; Secondly, FAT10 will influence the USP7 degradation with UPS; Third, we observed that competitive binding of Ub and FAT10 to USP7 attenuated the ubiquitination of USP7; Ultimately, our findings suggested that FAT10 overexpression raised the ubiquitination of USP7, whereas FAT10 silencing had the opposite effect. In addition, we found that FAT10 promoted CHK1-mediated G2/M arrest through USP7 in RTECs. Therefore, based on all of our results above, we established a working model of FAT10 promoting renal fibrosis (Figure 7). Under UUO injury, the levels of hypoxia in the obstructed kidneys increases, leading to the upregulation of FAT10 in RTECs; overexpression of FAT10 can stabilize USP7 by antagonizing the ubiquitination of USP7, resulting in increased levels of CHK1, which subsequently induces G2/M cell cycle arrest, triggering profibrotic TGF-β- and CTGF-mediated renal fibrosis.

In conclusion, renal fibrosis is a reliable predictor of poor prognosis and a common event in the course of development to the end-stage renal disease, regardless of the initial factor. Thus, it is imperative to explore the pathogenesis of renal fibrosis. In this study, we demonstrated that FAT10 accelerates renal fibrosis by stabilizing USP7 to promote CHK1-mediated G2/M arrest in RTECs. These findings suggest that FAT10 is a significantly mediator of renal fibrosis, and FAT10 is an underlying therapeutic target for treating the chronic renal fibrosis.

## Supplementary Materials

Supplementary Figures
