# Supplementary material for: Ubiquitin-like protein FAT10 promotes renal fibrosis by stabilizing USP7 to prolong CHK1-mediated G2/M arrest in renal tubular epithelial cells
Source: Aging (Albany NY). 2022 Sep 22;14(18):7527–46. doi: 10.18632/aging.204301 (PMC9550257; doi:10.18632/aging.204301)
Supplement: Supplementary Figures [file aging-14-204301-s001.pdf]

SUPPLEMENTARY FIGURES

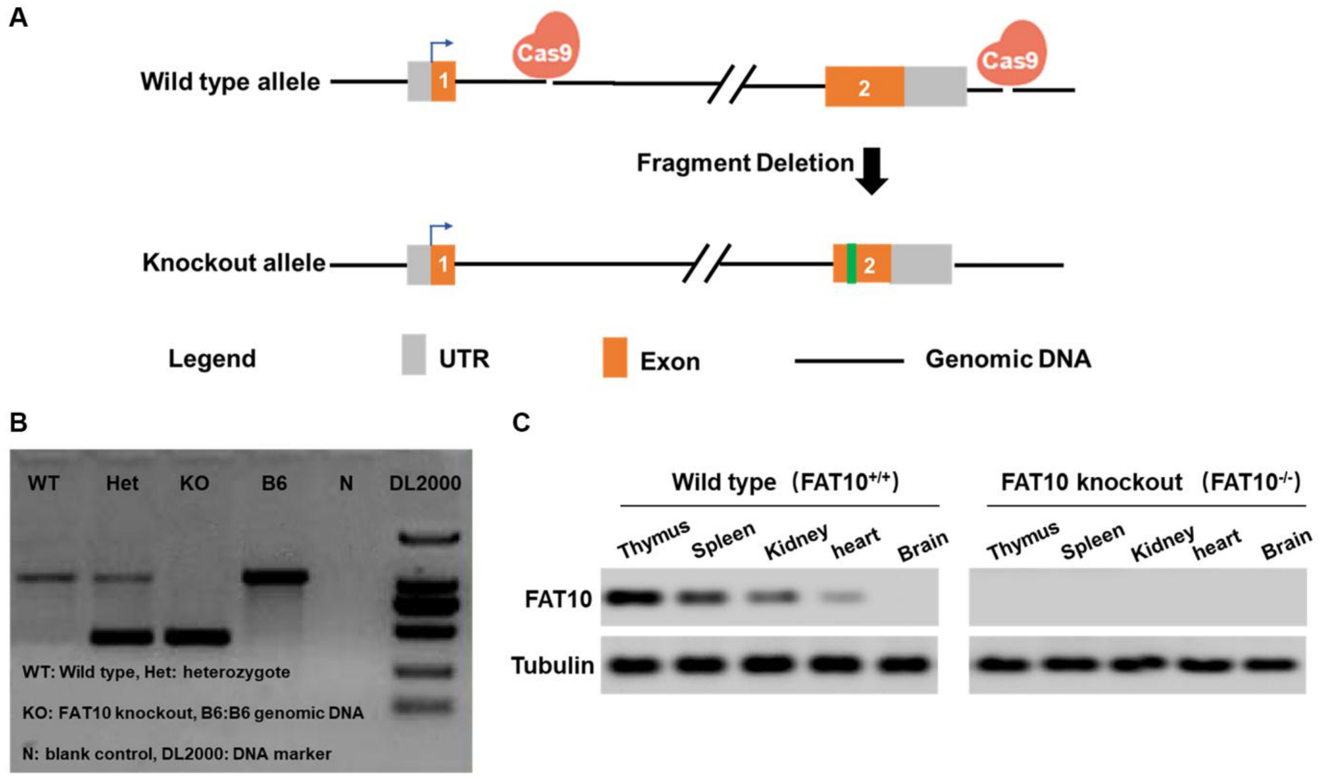

Supplementary Figure 1. Construction of FAT10 knockout mice. (**A**) Schematic diagram of FAT10-knockout mice (FAT10$^{-/-}$) generation by the CRISPR-Cas9 technique. (**B**) Genotypes of different mice were identified by special primers. (**C**) Western blot showing FAT10 protein levels in thymus, spleen, kidney, heart and brain from wild type (FAT10$^{+/+}$) and FAT10$^{-/-}$ mice.

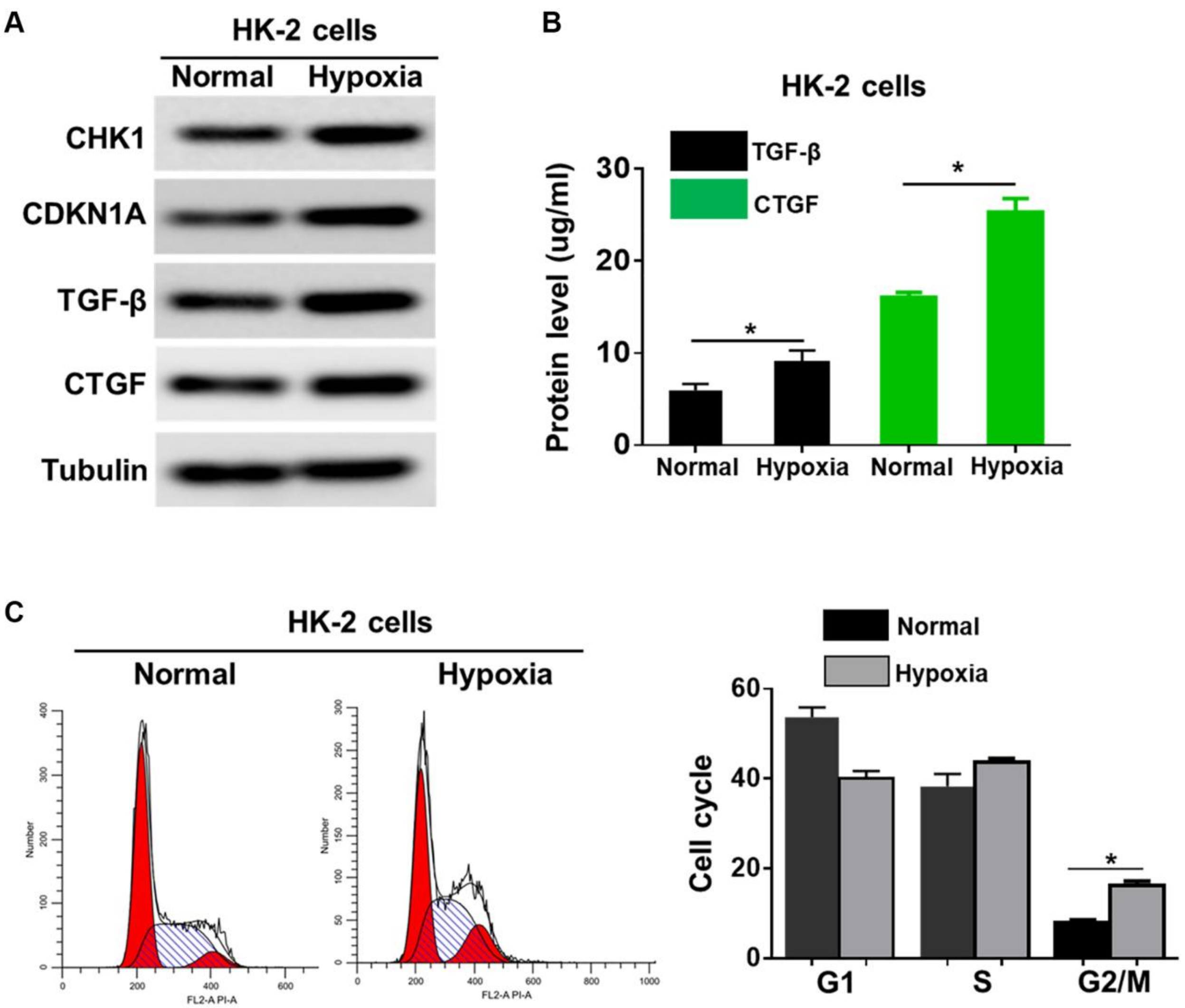

Supplementary Figure 2. CHK1 is involved in hypoxia induced G2/M arrest in HK-2 cells. (**A**) Western blotting showing the protein expression of CHK1, CDKN1A, TGF-β and CTGF in HK-2 cells following hypoxia injury. Tubulin was used as a loading control. (**B**) TGF-β and CTGF in the culture supernatants was measured in culture supernatants by ELISA assay. $^{*}P < 0.05$. (**C**) Detection for cell cycle of HK-2 cells following hypoxia injury. Results are expressed as peak diagram (left) and calculated distribution for cells in G0/G1, S and G2/M phases (right). $^{*}P < 0.05$.

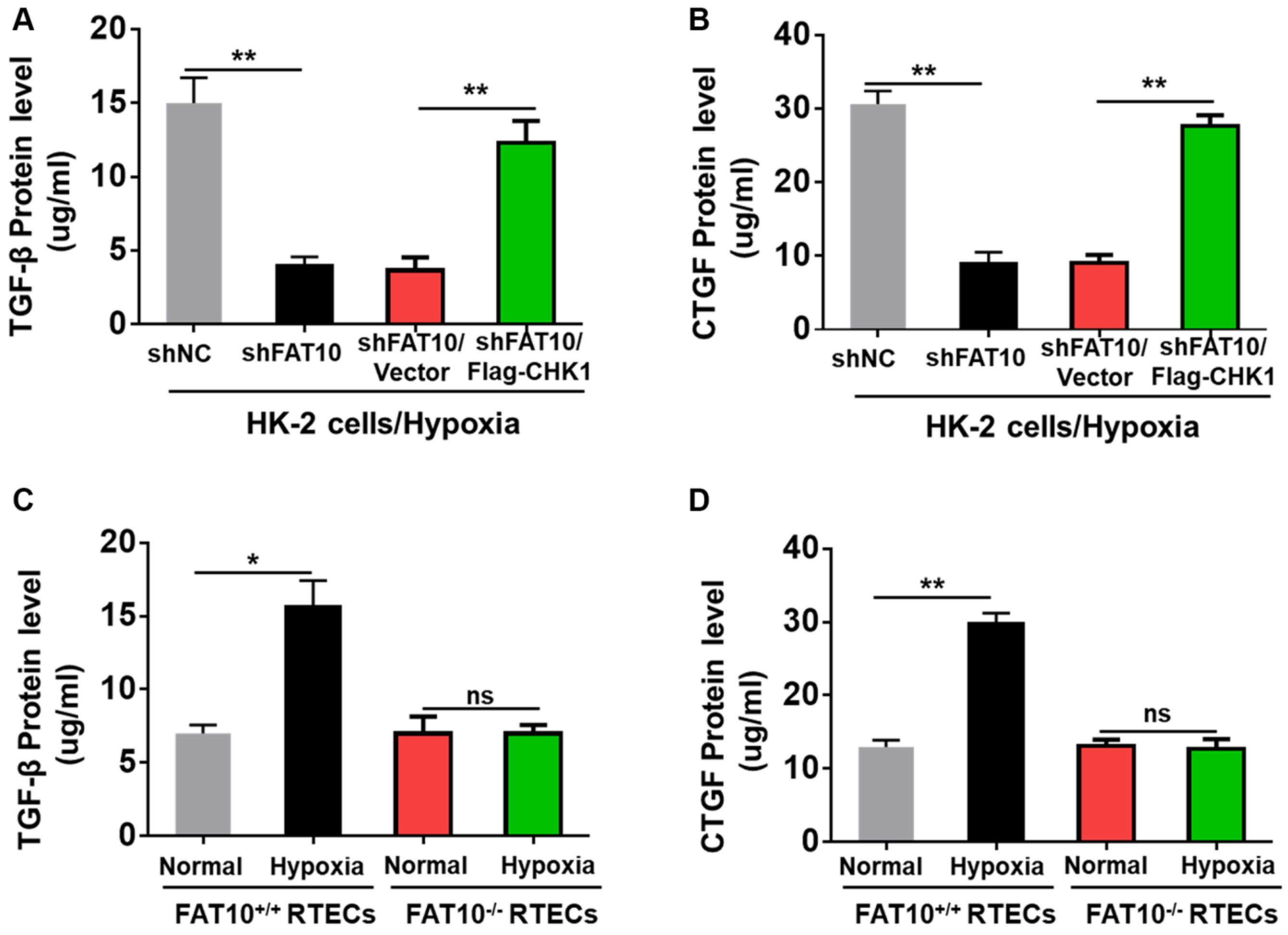

Supplementary Figure 3. (**A–D**) ELISA assay showing TGF-β and CTGF in the culture supernatants was measured in the different experimental groups. $^{*}P < 0.05$, $^{**}P < 0.01$. Ns indicates no significance.

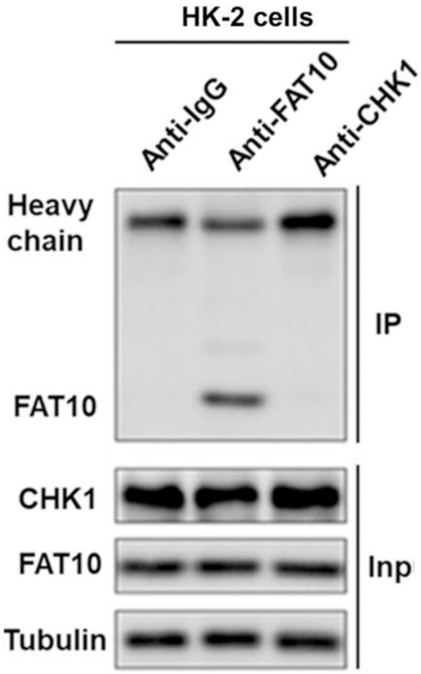

Supplementary Figure 4. Co-IP for endogenous FAT10 and CHK1 in HK-2 cells.

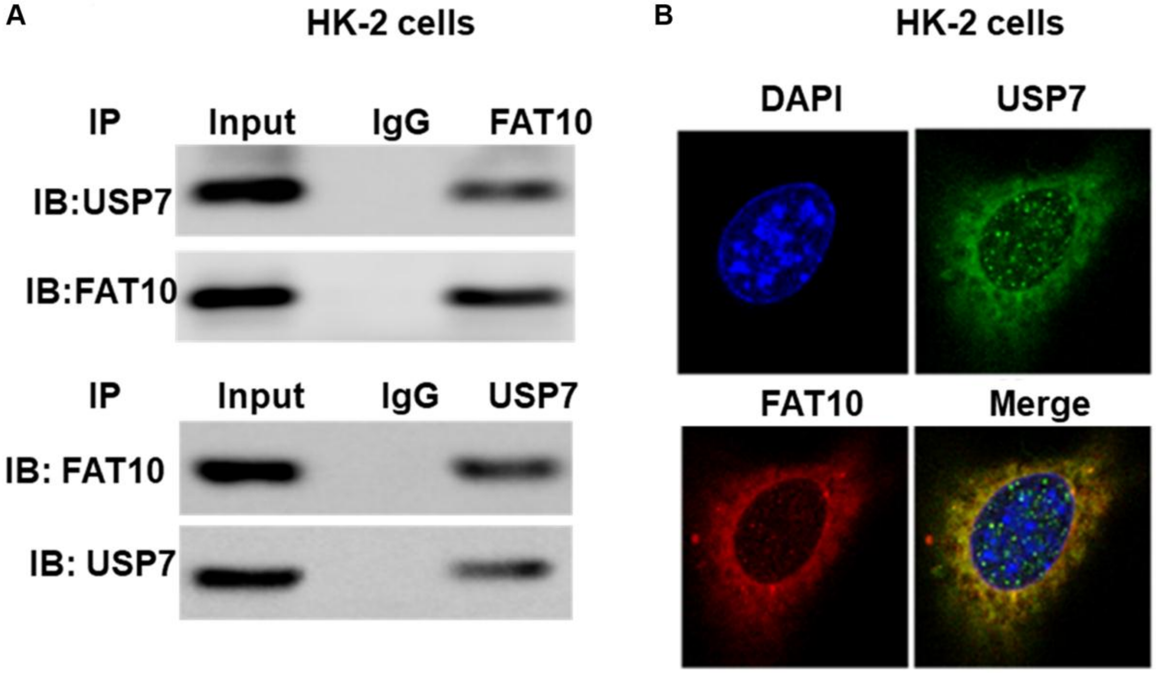

Supplementary Figure 5. FAT10 directly interacts with USP7. (**A**) Co-IP for endogenous FAT10 and USP7 in HK-2 cells. (**B**) Co-localization of FAT10 and USP7 in HK-2 cells; FAT10 (1:200) in red, USP7 (1:200) in green, and DAPI nuclear counterstaining in blue.

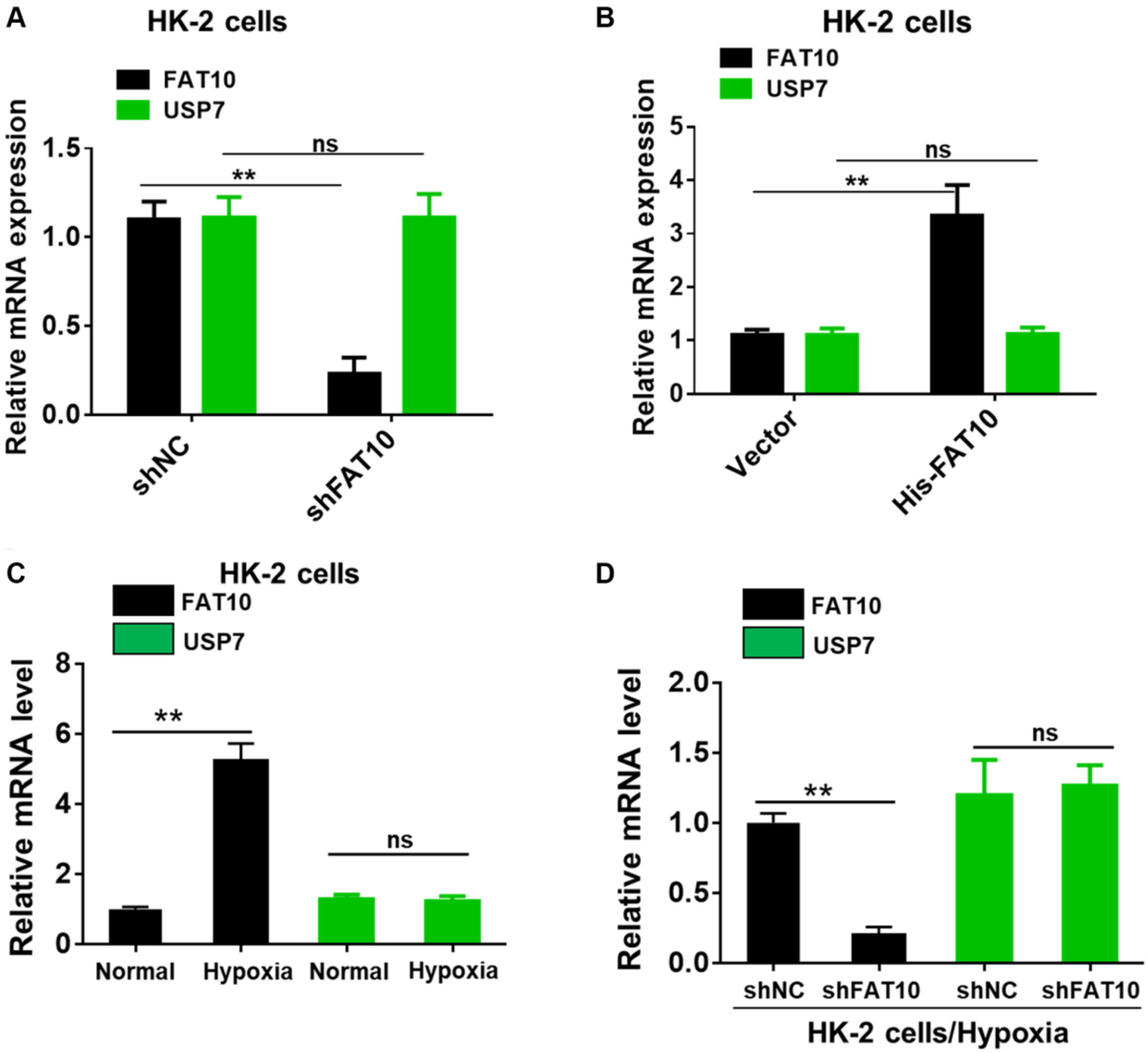

Supplementary Figure 6. The mRNA levels of FAT10 and USP7 were detected. (**A** and **B**) The mRNA levels of FAT10 and USP7 assessed by qRT-PCR in FAT10-overexpressing or FAT10-silenced HK-2 cells. $^{**}P < 0.01$. Ns indicates no significance. (**C** and **D**) The mRNA levels of FAT10 and USP7 assessed by qRT-PCR in HK-2 cells (**C**) or FAT10-silenced HK-2 cells (**D**) following treatment with hypoxia or without hypoxia. $^{**}P < 0.01$. Ns indicates no significance.

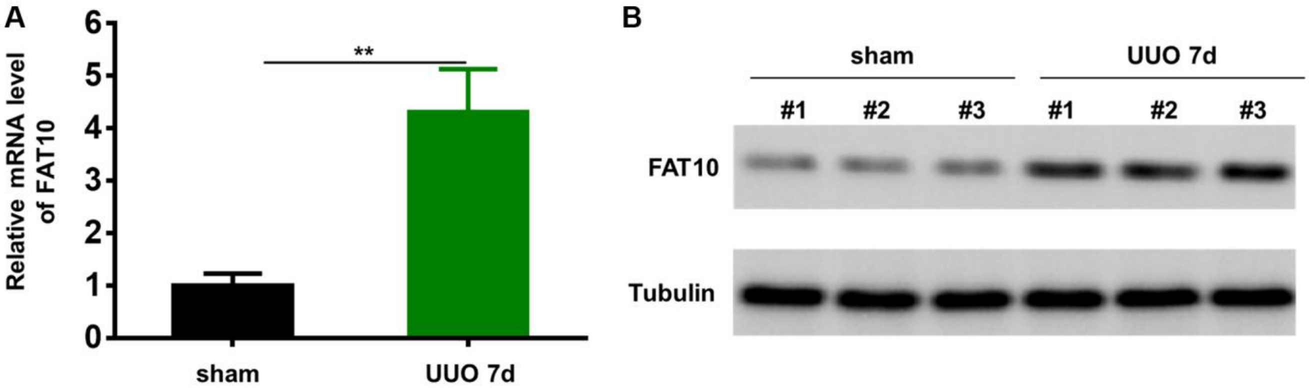

Supplementary Figure 7. Expression level of FAT10 is upregulated in obstructed kidney tissues. (**A**) Expression levels of FAT10 mRNA in kidney tissues of sham and UUO mice as detected by qRT-PCR. $N = 6$, $^{**}P < 0.01$ compared to the sham group. (**B**) Protein expression levels of FAT10 in kidney tissues of sham and UUO mice as detected by Western blotting. Tubulin was used as a loading control.

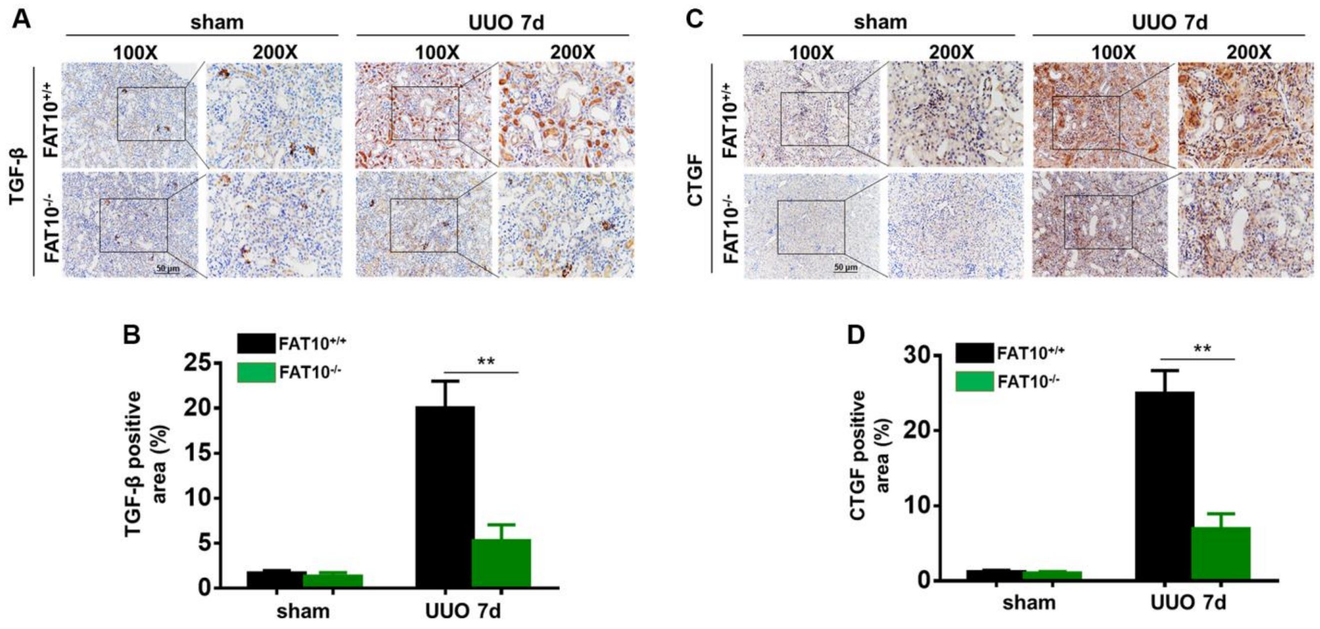

Supplementary Figure 8. Expression level of TGF-β and CTGF is upregulated in obstructed kidney tissues. (**A**) Immunohistochemistry of protein expression of TGF-β in obstructed kidneys from FAT10$^{+/+}$ and FAT10$^{-/-}$ mice subjected to either UUO or sham operation. (**B**) Bar graph shows quantification of areas of TGF-β positive cells; $^{**}P < 0.01$ versus FAT10$^{+/+}$ mice at the same time point; $n = 6$. (**C**) Immunohistochemistry of protein expression of CTGF in obstructed kidneys from FAT10$^{+/+}$ and FAT10$^{-/-}$ mice subjected to either UUO or sham operation. (**D**) Bar graph shows quantification of areas of CTGF positive cells; $^{**}P < 0.01$ versus FAT10$^{+/+}$ mice at the same time point; $n = 6$.
